# Add-On Effect of Hemagglutinating Virus of Japan Envelope Combined with Chemotherapy or Immune Checkpoint Inhibitor against Malignant Pleural Mesothelioma: An In Vivo Study

**DOI:** 10.3390/cancers15030929

**Published:** 2023-02-01

**Authors:** Kazuma Sakura, Masao Sasai, Soichiro Funaki, Yasushi Shintani, Meinoshin Okumura, Yasufumi Kaneda

**Affiliations:** 1 Respiratory Center, Osaka University Hospital, 2-15, Yamadaoka, Suita 565-0871, Osaka, Japan; 2 Division of Translational Research, Osaka University Hospital, 2-15, Yamadaoka, Suita 565-0871, Osaka, Japan; 3 Frontier of Regenerative Medicine, Graduate School of Medicine, Osaka University, 2-2, Yamadaoka, Suita 565-0871, Osaka, Japan; 4 National Toneyama Medical Center, 5-1-1, Toneyama, Toyonaka 560-8552, Osaka, Japan; 5 Administration Bureau, Osaka University, 1-1, Yamadaoka, Suita 565-0871, Osaka, Japan

**Keywords:** hemagglutinating virus of Japan envelope, pleural mesothelioma, PD-1, PD-L1, cis-diamminedichloroplatinum (II), CDDP, sugar chain, cytotoxic T cell, NK cells, immune checkpoint inhibitors

## Abstract

**Simple Summary:**

In this study, we report that the combination of existing therapeutic agents and hemagglutinating virus of Japan envelope (HVJ-E), a virus-inactivating agent, may be a more effective treatment agent for pleural mesothelioma, a refractory cancer for which no effective treatment has been established. We have already reported that HVJ-E, which activates antitumor immunity and induces apoptosis in cancer, can provide a different mechanism for effective treatment. In this report, the antitumor effect of HVJ-E in combination with chemotherapy or immune checkpoint inhibitors (ICIs) activated antitumor immunity in a mouse model of malignant pleural mesothelioma-bearing tumor and significantly enhanced antitumor efficacy compared to single-agent therapy. We report that the use of HVJ-E in combination with chemotherapy or ICIs, which are already used in clinical practice, may be more effective than single-agent therapy. However, further clinical studies are needed before the combination therapy can be used in clinical practice.

**Abstract:**

Malignant pleural mesothelioma (MPM) is a refractory tumor because most of the lesions are already disseminated at diagnosis. Previously, the main treatment for MPM was combination chemotherapy. However, recently, immune checkpoint inhibitors (ICIs) are also used. For better efficacy of MPM treatment, we focused on hemagglutinating virus of Japan envelope (HVJ-E), which activates antitumor immunity and induces tumor-specific cell death. In this paper, we aimed to determine whether HVJ-E as a single agent therapy or in combination with chemotherapy or ICIs is effective in MPM bearing mouse. We confirmed its antitumor efficacy in MPM-bearing mouse. HVJ-E significantly prolonged the survival of human MPM-bearing mouse compared to that of control mouse and when combined with CDDP. This efficacy was lost in NOD-SCID mouse, suggesting that activation of innate immunity by HVJ-E was related to the survival rate. HVJ-E also showed antitumor efficacy in murine MPM-bearing mouse. The combination of chemotherapy and HVJ-E caused a significant increase in cytotoxic T cells (CTLs) compared to chemotherapy alone, suggesting that not only innate immunity activated by HVJ-E but also the increase in CTLs contributed to improved survival. The combination of anti-PD-1 antibody and HVJ-E significantly prolonged the survival rate of murine MPM-bearing mouse. Further, HVJ-E might have exhibited antitumor effects by maintaining immunogenicity against tumors. We believe that HVJ-E may be a beneficial therapy to improve MPM treatment in the future.

## 1. Introduction

Malignant pleural mesothelioma (MPM) is an intractable tumor that is very difficult to cure [1]. It is also known as a silent time bomb because it develops decades after exposure to asbestos [2]. Chemotherapy has been the mainstay of treatment for MPM, and multidisciplinary treatment combining chemotherapy with surgery and radiotherapy has been used, but the results have not been satisfactory [3]. The reason for this is that most of the lesions are already disseminated in the thoracic cavity at the time of discovery, and radical treatment at the cellular level is impossible at this time [4]. However, as in other types of cancer, immune checkpoint inhibitors (ICIs; Anti PD-1 Antibody) have been reported to be effective in the treatment of MPM, and their use has been reported in combination with other type of ICIs (anti-CTLA-4 antibody) or chemotherapy as first-line, second-line, or later-line therapy [5,6]. In particular, ICIs are effective for non-epithelial MPMs, which do not respond to chemotherapy because of their relatively high expression of PD-L1, and can improve the overall response rate of MPMs [7]. Under these circumstances, combination therapy with ICIs and chemotherapy or other therapeutic reagents is expected to become the mainstay of treatment for MPMs, as in lung cancer treatment. High response rates have been actually reported in lung cancer treatment [8]. In this situation, we focused our attention on hemagglutinating virus of Japan envelope (HVJ-E) as a preparation that can be expected to be effective in combination with chemotherapy or ICIs to further improve treatment outcomes. HVJ-E exerts its therapeutic effect by enhancing the cancer immune cycle through activation of antigen-presenting cells such as dendritic cells and macropahges [9], killing cancer cells by cytotoxic T cells and Natural killer cells [10,11], and inducing M1- type macropahges through diverse immune activatinon effects [12,13,14]. In combination with chemotherapy and ICIs, HVJ-E is expected to have a high antitumor effect. HVJ-E is a non-viral preparation that exhibits antitumor effects by activating the immune system through pseudo-infection and by releasing molecules such as damage-associated molecular patterns by inducing tumor-specific cell death [15]. In this report, we conducted a basic study on the efficacy of HVJ-E in combination with chemotherapy or ICIs to confirm its efficacy.

## 2. Materials and Methods

### 2.1. Cell Lines and Mouse

The MSTO-211H human biphasic mesothelioma cell line, H2052 human sarcomatoid mesothelioma cell line [16], H2452 human epithelioid mesothelioma cell line, MeT-5A human mesothelium cell line that was not tumorigenic, and DU145 and PC3 human prostate cancer cell lines were obtained from American Type Culture Collection. The ACC-MESO-1 human sarcomatoid mesothelioma and ACC-MESO-4 human epithelioid mesothelioma cell lines were obtained from Riken Bioresource Research Center (Ibaraki, Japan) [17]. The EHMES-10 human biphasic mesothelioma cell line was kindly provided by Dr. Jitsuo Higaki and Dr. Hironobu Hamada, Ehime University [18]. AB22G2, which has a stable potential for grafting and spreading to pleural cavity was isolated from AB22 cells [19] after two successive cycles of in vivo selection procedures [20]. AB22 murine morphological epithelioid mesothelioma [21] was kindly provided by Dr. Cleo Robinson and Dr. Bruce WS Robinson, University of Western Australia. MSTO-H211, DU145, LNCap, EHEMS-10, ACC-MESO-1, ACC-MESO-4, and Met-5A were maintained in RPMI-1640 (Sigma-Aldrich, St. Louis, MO, USA) containing 10% fetal bovine serum, 1% (*v*/*v*) 100X nonessential amino acids, 1 mM sodium pyruvate, 2 mM L-glutamine, 50 μM 2-mercaptoethanol, 100 units/mL penicillin, and 100 μg/mL streptomycin. AB22G2, H2452, and H2052 were maintained in Dulbecco’s modified Eagle’s medium (Nacalai Tesque, Kyoto, Japan) containing the same additives as above.

BALB/c AJcl mouse and CB-17/Icr-scid/scidJcl mouse (age: 6–8 weeks) were obtained from CLEA Japan (Tokyo, Japan). NOG (NOD/Shi-scid, IL2Rγ null) mouse (age: 6 weeks) were obtained from Central Institute for Experimental Animals (Kanagawa Japan). All mice were housed in a temperature-controlled, pathogen-free room, and acclimatized for at least one week in the breeding room of the animal experimentation unit of Osaka University Graduate School of Medicine. In vivo experiments were performed in accordance with the approved protocols and guidelines of the Ethics Review Committee for Animal Experimentation of Osaka University Graduate School of Medicine (Osaka, Japan, #26-070-000).

### 2.2. Preparation of HVJ-E

HVJ (VR-105 parainfluenza Sendai/52 Z strain) was acquired from the American Type Culture Collection (Manassas, VA, USA) and prepared as previously described [22]. The HVJ seed solution was injected into embryonated eggs that were 10–14 days old and cultured in a 37 °C incubator for 3 days. After 3 days, chorioallantoic fluid was harvested from the eggs injected with HVJ. The purified virus (live HVJ) was inactivated by UV irradiation (189 mJ/cm^2^) to yield HVJ-E [22,23].

### 2.3. Labeling of PKH26 to HVJ-E

Cells (1.5 × 10^4^) were seeded into each well of an 8-well Lab-tek chamber and cultured overnight. Cells were incubated with HVJ-E with PKH26 at a concentration of 83 HAU per well for 2 h. After washing twice with complete culture medium, cells were fixed with 4% paraformaldehyde and were stained with 400 mM Hoechst 33342 (H1399; Thermo Fisher Scientific K.K. Tokyo, Japan) to stain the nucleus, and observed by a confocal microscope (Radiance 2100: Bio-Rad Japan, Tokyo, Japan).

### 2.4. Cytotoxic Assay

Five thousand cells, which settled in 100 μL per well of 96 well plate, were cultured for 24 h. Then, 67 HAU of HVJ-E was added into each well and incubated for 15 h. Tumor cells with HVJ-E were washed twice and culture medium was added into each well. CCK-8 solution was added into each well and cultured for 1.5 h, and each medium was added into each well of 96 well plate. The absorbance at 450 nm was measured according to manufacturer’s instructions (Cell Counting Kit-8; Dojindo, Kumamoto, Japan: https://www.dojindo.co.jp/manual/CK04e.pdf (accessed on 1 December 2022).

### 2.5. ELISpot Assay

AB22G2 subcutaneous tumor-bearing BALB/c mouse (mentioned as below) were treated with intratumoral injection with 1000 HAU of HVJ-E or 2 mg/kg of CDDP (Cis-diamminedichloroplatinum, Nippon Kayaku, Tokyo, Japan) 14 days after cell injection, followed by five additional treatments of HVJ-E every 3 days for a total of six treatments. The spleens were isolated from the mouse 5 days after the last treatment. Splenocytes were isolated from the spleens, filtered through a 40-μm mesh sieve, and hemolyzed in hemolysis buffer (Immuno-Biological Laboratories). AB22G2 cells were treated with mitomycin C (15 μg/mL) for 45 min. The splenocytes and mitomycin C-treated AB22G2 cells were mixed in a ratio of 10:1 and incubated at 37 degree in a humidified atmosphere of 5% CO_2_. After 48 h, nonadherent splenocytes were collected, and an ELISpot assay was performed using the Mouse IFN-gamma Development Module (R&D Systems, Minneapolis, MN, USA) and the ELISpot Blue Color Module (R&D Systems, Minneapolis, MN, USA). The numbers of IFN-gamma-secreting cells were subsequently counted.

### 2.6. Subcutaneous Tumor Model

One hundred thousand AB22G2 cells were subcutaneously administered into the right dorsal region of BALB/c mice, which were anesthetized with an intrapleural administration of a mixture of agents (midazolam, butorphanol tartrate, and medetomidine) [24]. Those mice were treated intratumorally (IT) with 2 mg/kg of CDDP and 1000 HAU of HVJ-E, CDDP alone, HVJ-E alone, or saline at day 14, followed by five additional IT of HVJ-E at days 3, 5, 7, 10, and 12. The subcutaneous tumor growth was monitored by measuring the three diameters of the tumor nodules, and the tumor volume was calculated using the following formula: volume (mm^3^) = 1/6 × π × d1 × d2 × d3 [25].

### 2.7. Orthotopic Pleural Tumor-Bearing Mouse Model

#### 2.7.1. MSTO-H211-Bearing CB-17/Icr-scid/scidJcl or NOG (NOD/Shi-scid, IL2Rγ Null) Model

Two million of MSTO-H211 in 50 μL of PBS with 100 μL of Matrigel (Matrigel Basement Membrane Matrix Phenol Red Free, #356237, Corning Japan, Tokyo, Japan) were administered into the murine parietal pleura confirming the ribs by incised skin of right chest with 30-gauge 1/2 needle (BD, Tokyo, Japan). Eight days after cell injection, 1000 HAU of HVJ-E or 3 mg/kg of CDDP was administrated into the right pleural cavity. Subsequently, 1000 HAU of HVJ-E was injected subcutaneously into the right anterior chest every 1 week.

#### 2.7.2. AB22G2-Bearing BALB/c AJcl Mouse Model

Two hundred thousand of AB22G2 cells in 50 μL of PBS with 100 μL of Matrigel were administered into the murine parietal pleura confirming the ribs by incised skin of right chest. Three days after the cell injection, 1000 HAU of HVJ-E or 2.5 mg/kg of CDDP was administrated into the right pleural cavity. Subsequently, 1000 HAU of HVJ-E was injected subcutaneously into the right anterior chest every 1 week.

#### 2.7.3. Add-On Effect of HVJ-E for ICIs (@PD-1 mAb., @PD-L1 mAb.) on AB22G2-Bearing Mouse Model

The same AB22G2-bearing mouse model as that mentioned in Section 2.7.2. was prepared. Four days after cell injection, 100 μg/100 μL of PD-1 (CD279, RMP1-14, BioLegend, San Diego, CA, USA) or PD-L1 (CD274, B7-H1, 10F.9G2, BioLegend, San Diego, CA, USA) monoclonal antibody was injected into the peritoneal cavity or 1000 HAU of HVJ-E was injected into the right pleural cavity. Subsequently, three subcutaneous injections of HVJ-E were administered every 3 days. These four injections of HVJ-E were considered as one cycle therapy, and this treatment was repeated two times maximally. Subsequently HVJ-E was injected subcutaneously into the right anterior chest every 3 days. During the entire treatment process, PD-1 or PD-L1 monoclonal antibody was administrated into the peritoneal cavity every four injections of HVJ-E (maximum four times administration).

#### 2.7.4. Add-On Effect of the Local Administration of HVJ-E for ICIs (@PD-1 mAb., @PD-L mAb.) on AB22G2-Bearing Mouse Model

In this AB22G2-bearing mouse model, the number of AB22G2 cells was modified to one hundred thousand. Three days after cell injection, 100 μg/100 μL of PD-1 monoclonal antibody was administrated into the peritoneal cavity, the second and third administration of PD-1 antibody was performed at 17 and 31 days after cell injection. Three days after cell injection, 1000 HAU of HVJ-E was injected into the right pleural cavity and subsequently three subcutaneous injections of HVJ-E were administered every 3 days. These four injections of HVJ-E were considered as one cycle therapy, and this therapy was repeated two times maximally, and subsequently HVJ-E was injected subcutaneously into the right anterior chest every 3 days (PD-1 with intrapleural and subcutaneous injection of HVJ-E group, or intrapleural and subcutaneous injection of HVJ-E group), or 1500 HAU of HVJ-E was injected subcutaneously into the right anterior chest and subsequently, the same dose of HVJ-E was injected subcutaneously every 3 days (PD-1 with subcutaneous injection of HVJ-E group, or subcutaneous injection of HVJ-E group).

### 2.8. Statistical Analyses

Student’s *t*-test was performed to determine statistical significance between the two groups. All results were expressed as the mean ± standard error of the mean (SEM). The differences between the groups in the survival experiment were determined by the Kaplan–Meier method and log-rank test. All values were considered statistically significant at a *p*−value of <0.05. All statistical analyses were carried out with EZR version 1.40 (Saitama Medical Center, Saitama, Japan), which is a graphical user interface for R commander version 2.5-1/R version 3.5.2 (The R Foundation for Statistical Computing, Vienna, Austria) [26]. The probability value of <0.05 was considered statistically significant.

## 3. Results

### 3.1. Affinity of HVJ-E to Mesothelioma and Prostate Cancer Cell Lines

**Figure 1:**
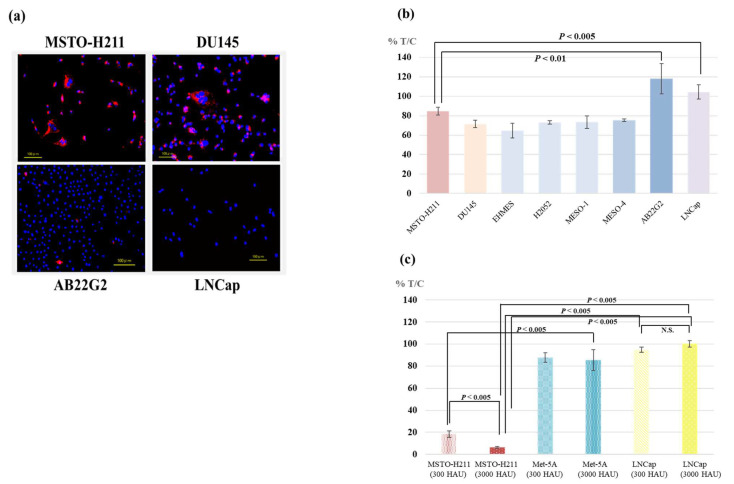
(**a**) The Affinity of HVJ-E to mesothelioma and prostate cancer cell lines. MSTO-H211, AB22G2, DU145, and LNCap cells were incubated with PKH26-labeled HVJ-E for 2 h, and washing. (**b**) The cytotoxicity of HVJ-E against various tumor cell lines. Cells were treated with HVJ-E (67 HAU). Fifteen hours after HVJ-E treatment, cell survival was assessed by Water-Soluble Tetrazolium assay. Each experiment was repeated 3 times with similar results. HVJ-E showed significant cytotoxicity against human mesothelioma cell lines than human prostate hormone-resistant cell LNCap and murine mesothelioma cell AB22G2. MSTO vs. AB22G2 *p* < 0.01, MSTO vs. LNCap *p* < 0.005, DU145 vs. LNCap *p* < 0.001, DU145 vs. AB22G2 *p* < 0.01, MSTO vs. DU145 *p* < 0.05 (**c**) The cytotoxicity of HVJ-E against MSTO-H211, Met-5A and LNCap. The same as above procedure of (**b**) was done except the applied quantity of HVJ-E (300 or 3000 HAU). Twenty-four hours after HVJ-E treatment, cell survival was measured by the same procedure as in (**b**). Despite the dose-dependent manner cytotoxicity of HVJ-E against MSTO-H211, HVJ-E showed a little cytotoxicity and no dose dependency against LNCap and Met-5A compared to MSTO-H211 even at high doses of HVJ-E. “N.S.” means not significant.

PKH26-labelled HVJ-E preferentially bound to MSTO-H211 and DU145 compared to AB22G2 and LNCap tumor cells. The preferential binding of HVJ-E to MSTO-H211 was similar to the binding of HVJ-E to DU145 (Figure 1a).

### 3.2. Cytotoxicity of HVJ-E against Various Tumor Cell Lines

The cytotoxicity of HVJ-E (67 HAU) to AB22G2 was not observed as well as to LNCap. While, HVJ-E showed significantly high cytotoxicity to five human mesothelioma cell lines and DU145 cells than AB22G2 and LNCap (Figure 1b).

The cytotoxicity of HVJ-E to MSTO-H211 showed a dose-dependent manner, while there was no cytotoxicity to LNCap and Met-5A, even with 4.5- and 45-fold higher doses of HVJ-E than in the experiment as shown in Figure 1b (Figure 1c).

### 3.3. Survival of Human Malignant Pleural Mesothelioma-Bearing CB-17/SCID Mouse or NOG Mouse

**Figure 2:**
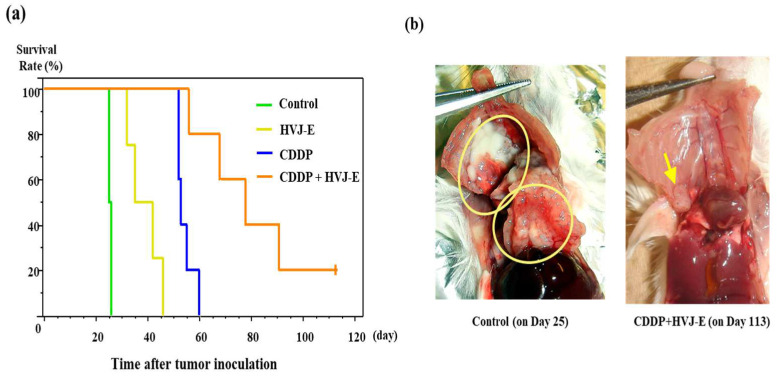

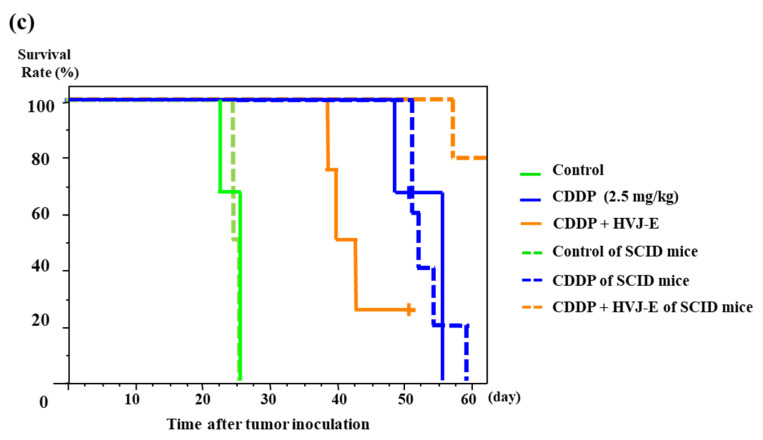
Antitumor effect of HVJ-E and CDDP against human malignant pleural mesothelioma-bearing mouse. Each in vivo experiment was repeated two times. (**a**) The survival rate of mouse after MSTO-H211 cells injection (*n* = 5). Eight days after the cell injection, 1000 HAU of HVJ-E or 3 mg/kg of CDDP was administrated into the right pleural cavity. Subsequently, 1000 HAU of HVJ-E was injected subcutaneously into the right anterior chest every 1 week. Differences between the groups in the survival experiment were determined by the Kaplan-Meier log-rank test. The mean survival time of mice that were treated with CDDP and HVJ-E concurrently was significantly prolonged compared with that of mouse treated with HVJ-E alone (*p* < 0.005), CDDP alone (*p* < 0.005), and 0.9% NaCl solution as a control (*p* < 0.005). The mean survival time of mouse treated with CDDP alone was significantly prolonged compared with that of mouse treated with HVJ-E alone (*p* < 0.01), and control group (*p* < 0.01), and that of mouse treated with HVJ-E alone was significantly prolonged compared with that of the control (*p* < 0.01). (**b**) The macroscopic findings of the pleural cavity of human pleural mesothelioma-bearing mouse. The right panel shows huge tumor masses disseminated in the pleural cavity of mouse treated with control 25 days after tumor cells implantation. Yellow circles indicate areas where the large tumor tissue was visibly present. The left panel shows controlled small tumor in the pleural cavity of mouse treated with CDDP and HVJ-E concurrently 113 days after tumor cells implantation. The yellow arrow indicates the smaller size of the tumor compared to that of the control mouse. (**c**) The survival rate of NOG mouse after MSTO-H211 cells injection (*n* = 4). Eight days after cell injection, 1000 HAU of HVJ-E or 2.5 mg/kg of CDDP was administrated into the right pleural cavity. Subsequently, 1000 HAU of HVJ-E was injected subcutaneously into the right anterior chest every 1 week. Differences between the groups in the survival experiment were determined by the Kaplan-Meier log-rank test. The mean survival times of NOG mice that were treated with CDDP and HVJ-E concurrently (orange solid line) and treated with CDDP alone (blue solid line) were significantly prolonged compared with that of mouse treated with control (green solid line) (*p* < 0.05). However, there was no significant difference between the mean survival time of mice that were treated with CDDP and HVJ-E concurrently and CDDP alone (*p* = 0.1831). Each dashed line shows the mean survival time of SCID mouse (**a**). The survival times of SCID mice and NOG mice were almost identical except for CDDP and HVJ-E concurrently. However, the mean survival time of NOG mouse that was treated with CDDP and HVJ-E (orange solid line) was markedly shortened compared to that of SCID mouse (orange dashed line).

In the CB-17/SCID mouse model, single-agent of CDDP or HVJ-E also significantly prolonged the survival time compared with controls, and the combination of both agents showed significant combined effect compared with single agent of CDDP or HVJ-E (Figure 2a). Macroscopic findings showed that controlled small tumor in the pleural cavity of mouse treated with CDDP and HVJ-E concurrently was detected 113 days after tumor cells implantation compared to huge tumor masses disseminated in the pleural cavity of mouse treated with saline (control mouse) 25 days after tumor cells implantation (Figure 2b). At 8 days after tumor cell implantation, tumors filled the pleural cavity (Figure S1). The mean survival time of NOG mice that were treated with CDDP alone and treated with CDDP and HVJ-E was significantly prolonged compared to the control. However, in NOG mouse, there was no synergistic effect of HVJ-E that was observed in SCID mouse treated with CDDP observed in SCID mouse (Figure 2c).

### 3.4. Survival of Murine Malignant Pleural Mesothelioma-Bearing Mouse

**Figure 3:**
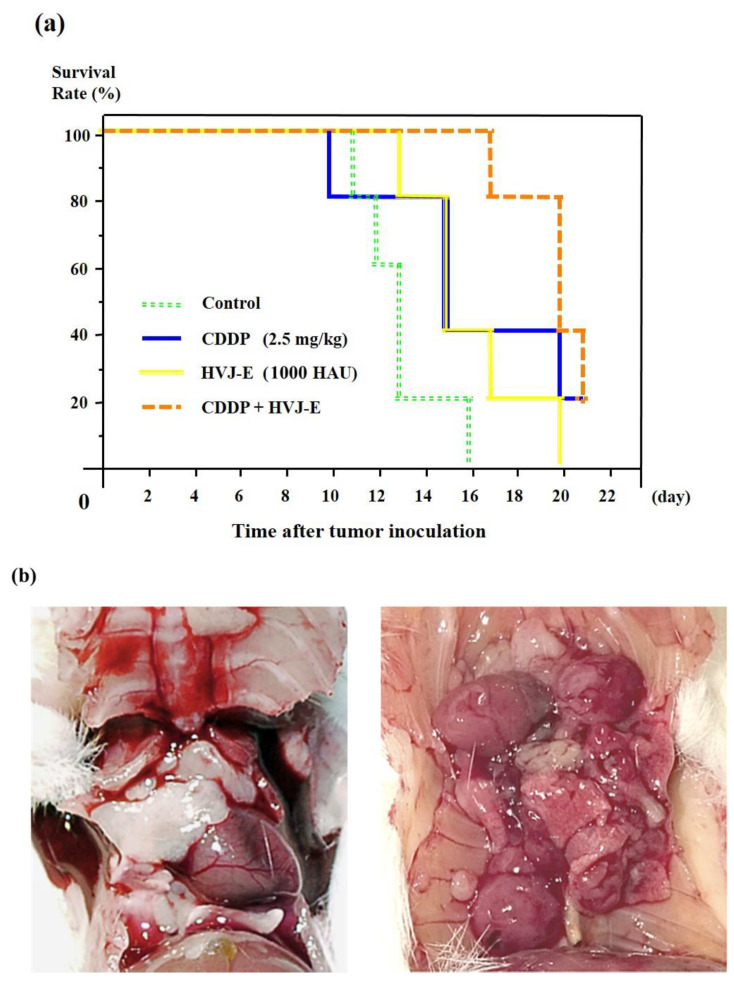
Antitumor effect of HVJ-E and CDDP against murine malignant pleural mesothelioma-bearing mouse. (**a**) The survival rate of mouse after AB22G2 cells injection. Three days after cell injection, 1000 HAU of HVJ-E or 2.5 mg/kg of CDDP was administrated into the right pleural cavity (*n* = 5). Subsequently, 1000 HAU of HVJ-E was injected subcutaneously into the right anterior chest every 1 week. Differences between the groups in the survival experiment were determined by the Kaplan-Meier log-rank test. The mean survival time of mice that were treated with CDDP and HVJ-E concurrently was significantly prolonged compared with that of mouse treated with HVJ-E alone (*p* < 0.05) and control (*p* < 0.005). Meanwhile, there was no significant difference in the mean survival of mice that were treated with CDDP and HVJ-E concurrently and that treated with CDDP alone. There was also no significant difference between control group and HVJ-E alone or CDDP alone group. (**b**) Macroscopic findings of the pleural cavity of murine MPM-bearing mouse with control, or CDDP with HVJ-E. The macroscopic findings of the pleural cavity of human pleural mesothelioma-bearing mouse. Right photo shows huge tumor masses disseminated in the pleural cavity of mouse treated with control 10 days after tumor cells implantation. Left photo shows controlled small tumor in the pleural cavity of mouse treated with CDDP and HVJ-E concurrently 21 days after tumor cells implantation.

Instead of heterologous mouse model, an allogeneic orthotopic mesothelioma bearing mouse model was used. In this model, the innate and the adaptive immune system of mice were not compromised, however HVJ-E had no antitumor effect against AB22G2 that expressed few HVJ-E receptor. The combination of CDDP and HVJ-E concurrently significantly prolonged the survival time of the mouse compared to HVJ-E alone or control group (Figure 3a).

Macroscopic findings showed that controlled small tumor in the pleural cavity of mouse treated with CDDP and HVJ-E concurrently was detected 21 days after tumor cells implantation compared to huge tumor masses disseminated in the pleural cavity of mouse treated with saline (control mouse) 10 days after tumor cell implantation (Figure 3b).

### 3.5. AB22G2 Specific INF-γ Response on AB22G2 Subcutaneous Tumor Model Treated by HVJ-E and CDDP

**Figure 4:**
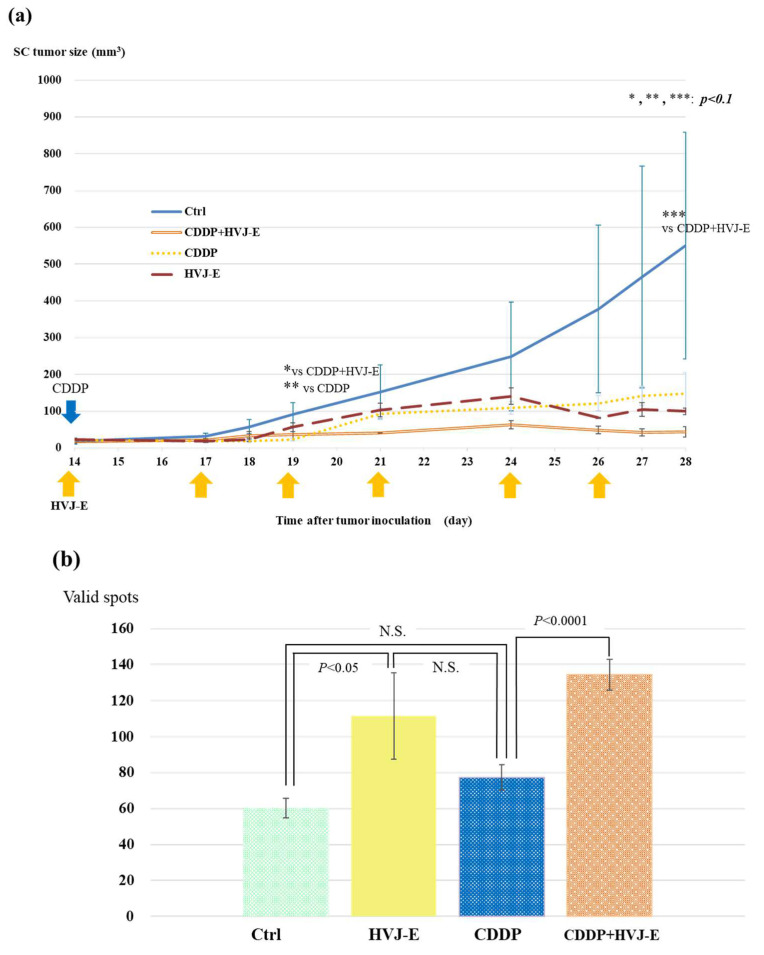
HVJ-E and CDDP significantly suppressed AB22G2 subcutaneous tumor growth and induced an AB22G2 specific INF-γ response. AB22G2 cells were subcutaneously implanted into right dorsal region of BALB/c mouse at day 0. Those mice were treated intratumorally with 2 mg/kg of CDDP and 1000 HAU of HVJ-E, CDDP alone, HVJ-E alone, or saline at day 14, followed by five additional intratumoral injection of HVJ-E at days 3, 5, 7, 10, and 12 (*n* = 4). (**a**) At 28 days after tumor cell injection, the tumor growth of the mouse treated with CDDP and HVJ-E concurrently was inhibited significantly compared with that of HVJ-E alone and CDDP alone. However, there was not a significant difference between that of CDDP and HVJ-E concurrently and the control group, because the tumor volume of the control group varied widely. The means ± SD of tumor volumes calculated from the diameter of the tumor mass are presented (*n* = 4 per group). (**b**) Tumor-specific INF-γ-secreting T cells were measured with an ELISpot assay. Values are stated as the mean ± SD. The IFN-γ enzyme-linked immune absorbent spot (ELISpot) assay revealed that mouse treated with CDDP and HVJ-E concurrently had significantly increased IFN-γ producing splenocytes compared with CDDP alone and the control group, and HVJ-E mouse treated with HVJ-E alone had significant difference compared with the control group (*p* < 0.05). “N.S.” means not significant.

To prove the add-on effect of HVJ-E against CDDP treatment, we used AB22G2 subcutaneous tumor-bearing mouse model that was treated with the intratumoral injection of HVJ-E, with the intraperitoneal administration of CDDP concurrently. At 28 days after tumor cell injection, the tumor growth of the mouse treated with CDDP and HVJ-E concurrently was inhibited significantly compared with that of HVJ-E alone or CDDP alone. However, it was not a significant difference between that of CDDP and HVJ-E concurrently and control group, because the tumor volume of the control group varied widely (Figure 4a). Then, Tumor-specific INF-γ-secreting T cells were measured with an ELISpot assay. The IFN-γ enzyme-linked immune absorbent spot (ELISpot) assay revealed that mouse treated with CDDP and HVJ-E concurrently had significantly increased IFN-γ producing splenocytes compared with CDDP alone and the control group (Figure 4b). In this experiment, there was no significant difference between CDDP and HVJ-E concurrently and HVJ-E alone group.

### 3.6. Survival of Murine Malignant Pleural Mesothelioma-Bearing Mouse Treated with HVJ-E and PD-1 (CD279) Monoclonal Antibody

**Figure 5:**
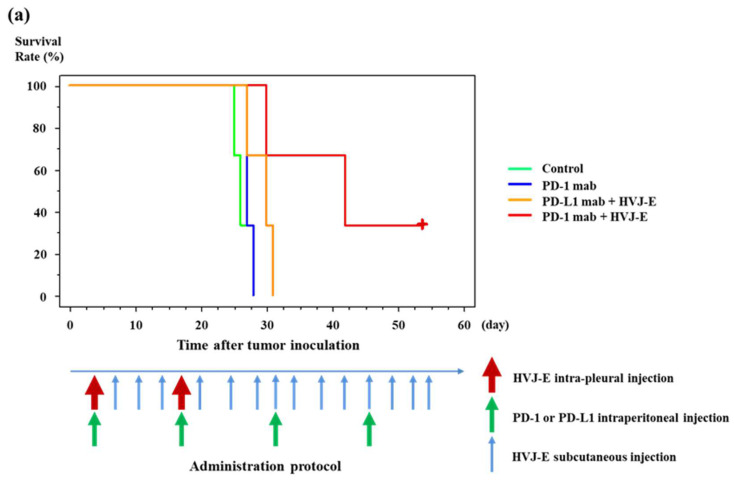

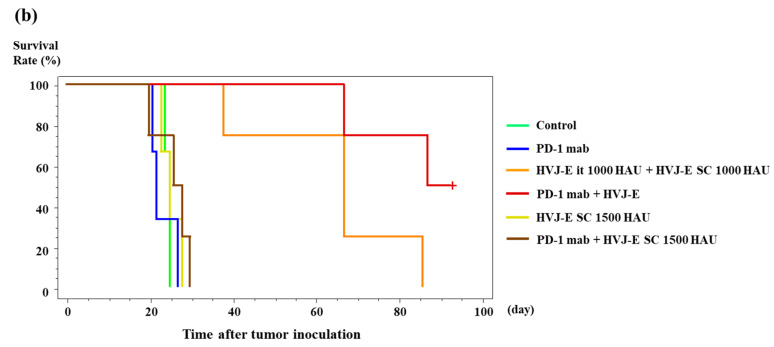
Survival of murine malignant pleural mesothelioma-bearing mouse treated with HVJ-E and PD-1 (CD279) monoclonal antibody. Each in vivo experiment was repeated two times. (**a**) Four days after AB22G2 cells injection, anti-PD-1 mAb or anti-PD-L1 mAb was injected into the peritoneal cavity (*n* = 3), or 1000 HAU of HVJ-E was injected into the right pleural cavity (*n* = 3) and subsequently three subcutaneous injections of HVJ-E was administered every 3 days (*n* = 3). These four injections of HVJ-E were considered as one cycle therapy, and this treatment was repeated two times maximally. Subsequently, HVJ-E was injected subcutaneously into the right anterior chest every 3 days. During the entire treatment process, anti-PD-1 or anti-PD-L1 mAb was administrated into the peritoneal cavity every four injections of HVJ-E (maximum 4 times administration). Differences between the groups in the survival experiment were determined by the Kaplan-Meier method and log-rank test. The mean survival time of mice that were treated with HVJ-E and anti-PD-1 mAb concurrently was significantly prolonged compared with that of anti-PD-1 mAb and the control group (*p* < 0.05). However, there was no significant difference between HVJ-E and PD-1 mAb concurrently or HVJ-E and PD-L1 mAb concurrently (*p* = 0.1341). (**b**) Three days after AB22G2 cell injection, anti-PD-1 mAb was administrated into the peritoneal cavity, the second and third administration of anti-PD-1 mAb was performed at the same day of described below the fifth and ninth administration of HVJ-E (anti-PD-1 group and anti-PD-1 with HVJ-E groups) (*n* = 4). Three days after cell injection, 1000 HAU of HVJ-E was injected into the right pleural cavity and subsequently three subcutaneous injections of HVJ-E were administered every 3 days. These four injections of HVJ-E were considered as one cycle therapy, and this therapy was repeated 2 times maximally, and subsequently HVJ-E was injected subcutaneously into the right anterior chest every 3 days (anti-PD-1 with intrapleural and subcutaneous injection of HVJ-E group, or intrapleural and subcutaneous injection of HVJ-E group), or 1500 HAU of HVJ-E was injected subcutaneously into the right anterior chest and subsequently, the same dose of HVJ-E was injected subcutaneously every 3 days (anti-PD-1 with subcutaneous injection of HVJ-E group, or subcutaneous injection of HVJ-E group). Differences between the groups in the survival experiment were determined by the Kaplan-Meier method and log-rank test. The mean survival time of mice that were treated with anti-PD-1 with intrapleural and subcutaneous injection of HVJ-E was significantly prolonged compared to subcutaneous injection of relatively high dose HVJ-E (*p* < 0.05), anti-PD-1 mAb (*p* < 0.05), anti-PD-1 mAb with subcutaneous injection of relatively high dose HVJ-E concurrently (*p* < 0.01), or the control group (*p* < 0.05), and intrapleural injection and subcutaneous injection of HVJ-E (*p* < 0.05), respectively.

The mean survival time of mouse treated with HVJ-E with anti-PD-1 mAb concurrently was significantly prolonged compared with that of anti-PD-1 monoclonal antibody alone or the control group. There was no significant difference between HVJ-E with PD-1 mAb concurrently and HVJ-E with PD-L1 mAb concurrently (Figure 5a), however there was a tendency towards the mouse treated with HVJ-E with PD-1 mAb concurrently to live longer than those treated with HVJ-E with PD-L1 mAb concurrently. The mean survival time of mice that were treated with anti-PD-1 with intrapleural and subcutaneous injection of HVJ-E was significantly prolonged compared to continuous subcutaneous injection of high dose HVJ-E, anti-PD-1 mAb, anti-PD-1 mAb with sub-cutaneous injection of high dose HVJ-E concurrently, or the control group (Figure 5b). Interestingly, the survival time of mouse treated with intrapleural administration of anti-PD-L1 mAb and continuous subcutaneous injection of HVJ-E was significantly prolonged compared to that treated with intrapleural administration of anti-PD-L1 mAb alone or control group (Figure S2). There was no significant difference between the survival time of mouse treated with anti-PD-1 mAb and PD-1 mAb with continuous subcutaneous injection of HVJ-E (Figure S3).

## 4. Discussion

### 4.1. Affinity and Cytotoxicity of HVJ-E against Various Cell Lines

HVJ-E bind to MSTO-H211 and other human mesothelioma cell lines preferentially compared to AB22G2 tumor cells. This preferential binding of HVJ-E is similar to the DU145 and LNCap human prostate cancer cell lines [11]. The preferential binding of HVJ-E to DU145 compared to LNCap has been already reported [11]. DU145 expressed large amounts of certain gangliosides which were the receptors of HVJ-E, while LNCap expressed little of them [11]. Various human mesothelioma cell lines expressed large amounts of such gangliosides, while murine mesothelioma cell AB22G2 did not express them (Figure S4).

HVJ-E showed significant cytotoxicity to various human mesothelioma and DU145 cells compared to AB22G2 and LNCap (Figure 1b). The cytotoxicity of HVJ-E to MSTO-H211 showed a dose-dependent manner, but no dependency to LNCap was observed (Figure 1c). The reason why there was no cytotoxicity of HVJ-E to LNCap and AB22G2 was that the HVJ-E did not bound to LNCap and AB22G2 (Figure 1a). HVJ-E showed the cytotoxicity to various mesothelioma cell lines but not to Met-5A human immortalized mesothelial cell as well as measles virus did. Met-5A expressed significantly lower level of CD46, the cellular receptor of measles, than most mesothelioma cells [27]. Therefore, it was supposed that Met-5A expressed only a few receptors of HVJ-E similar to measles. In addition, the receptors for HVJ, which is pathogenic to mice, were distributed only in the respiratory epithelium in mouse [28,29]. Thus, the intrapleural administration of HVJ-E was performed without worry of the injury of pleura due to HVJ-E.

### 4.2. Antitumor Effects of HVJ-E

Ten days after intratumoral inoculation of cells, tumor extension into the thoracic cavity and invasion into the epicardium and contralateral thoracic cavity were observed in a CB-17/SCID mouse model of intratumoral seeding of the human mesothelioma cell line MSTO-H211 (Figure S1). Since SCID mice were used in this treatment model that do not have cytotoxic T cells, the direct cytotoxicity of MSTO-H211 and the cytotoxicity induced by activation of the innate immune system and CDDP may have had a synergistic effect. In the control group, MSTO-H211 had disseminated and spread throughout the thoracic cavity on day 25 after cell administration, while in the combination treatment group, tumors formed and remained at the site where the cells were first inoculated even on day 113. Such impaired tumor development and growth may correspond to tumor dormancy, which is often observed in immunotherapy in clinical practice (Figure 2b). There was no synergistic effect of HVJ-E with CDDP in NOG mouse. In SCID mouse model, the cooperation of the direct antitumor effect of HVJ-E against MSTO-H211, the antitumor effect by the activation of NK cells, macrophages and the infiltration of these cells formed a synergistic effect, while in NOG mouse model, the synergistic effect of HVJ-E was completely lost due to the lack of innate immune system, although the direct antitumor effect was maintained. In this orthotopic model of human mesothelioma, the innate immunity contributes to the antitumor effect markedly. The significance of the combined use of intrapleural and subcutaneous administration of HVJ-E was investigated. In the MSTO-H211 tumor-bearing model, combining intrapleural administration of HVJ-E with subcutaneous administration of HVJ-E was suggested to enhance the antitumor effect of HJV-E, with a trend toward greater antitumor effect with more frequent subcutaneous administration of HVJ-E (Figure S5). In combination with CDDP, the addition of only subcutaneous administration of HVJ-E did not show synergistic effect, suggesting that intrapleural administration of HVJ-E is necessary (Figure S6).

### 4.3. Investigating the Effects on the Acquired Immunity

In the mouse mesothelioma cell line AB22G2 tumor model, the combination treatment group showed significantly prolonged survival compared to the HVJ-E alone group and the control group, but not significantly different from the CDDP alone group. On the other hand, the HVJ-E alone group and the CDDP alone group showed no significant difference from the control group, suggesting that the antitumor effect may not be sufficient for AB22G2 tumor-bearing mouse under the current conditions (Figure 3a). This model is an allogeneic transplant model, and the survival rate of the controls averaged a little more than 12 days, which is much more disabling to mouse than the 25 days of the SCID mouse model. The thoracic cavity was filled with large white tumors on day 10 after cell inoculation, whereas the thoracic cavity of the combination group was filled with only scattered small tumors on day 21 after cell inoculation, indicating the efficacy of the combination therapy (Figure 3b). This model is an orthotopic transplant model, which has a faster cell proliferation rate than the xenograft model, confirming that it is possible to control survival by reducing the number of inoculated cells (data not shown). In this model of intense proliferation, the combination of CDDP and HVJ-E showed no significant difference compared to CDDP, but did significantly prolong survival compared to the control and HVJ-E groups. First, it should be noted that only three HVJ-E cycles were performed compared to the MSTO-H211 model. It is important to note that this model has only three HVJ-E inoculations compared to the model of MSTO-H211, even though this model has a higher intensity of antitumor immunity added to the effect of the anticancer drug. Also, the fact that a significant antitumor effect is observed by adding the anticancer drug where only three inoculations of HVJ-E are possible, and that there is no significant difference between the anticancer drug only and the combined group, indicates that the antitumor effect of the anticancer drug is higher compared to that of HVJ-E.

### 4.4. Effects of HVJ-E on Cytotoxic T Cells

The following experiments on the effect of HVJ-E on CTL were performed using the AB22G2 subcutaneous tumor model. In the subcutaneous tumor model, HVJ-E was administered continuously intratumorally instead of intratumorally and subcutaneously, which requires interpretation. In a subcutaneous tumor model, tumor growth was significantly inhibited in the combination group compared to the control group (Figure 4a).

In addition, when the secretory capacity of IFN-γ was checked and the degree of potentiation of tumor-specific CTLs was confirmed, the combination group showed a significant potentiation of CTLs compared to the CDDP alone group. On the other hand, there was no significant difference between the addition of CDDP to HVJ-E, and the HVJ-E alone group which enhanced CTL significantly compared to control group. (Figure 4b). HVJ-E not only activates innate immunity in the MSTO-H211 experimental system, but also the acquired immune system in mesothelioma models. To clarify the mechanism of combination therapy with CDDP and HVJ-E, the immunogenicity of HVJ-E was confirmed. IFN- enzyme-linked immune absorbent spot (ELISpot) assay revealed that HVJ-E and CDDP-treated mouse had significantly increased IFN-γ-producing splenocytes compared to other treatment groups.

### 4.5. Investigate the Effect of Combination with ICIs

We investigated the therapeutic effect of PD-1 antibody in combination with HVJ-E in an AB22G2 orthotopic transplantation model. On the other hand, the group that received the initial intratumoral administration of HVJ-E followed by weekly subcutaneous administration (HVJ-E ip + sc) and the group that received the PD-1 antibody (PD-1 mAb + HVJ-E ip + sc) were significantly higher than the control, the group that received only subcutaneous administration without initial intratumoral administration of HVJ-E (HVJ-E sc alone), PD-1 antibody alone (PD-1 mAb alone), or PD-1 antibody plus HVJ-E subcutaneously (PD-1 mAb + HVJ-E sc) (*p* < 0.05). PD-1 mAb + HVJ-E ip + sc vs. PD-1 mAb + HVJ-E sc alone, PD-1 mAb + HVJ-E ip + sc showed significantly prolonged survival, suggesting that HVJ-E ip is necessary for the antitumor effect in this model (*p* < 0.01, *p* = 0.0067). PD-L1 antibody was also administered intraperitoneally and in combination with HVJ-E initial intratumoral and continuous subcutaneous administration (PD-L1 mAb + HVJ-E ip + sc), but there was no clear survival benefit compared with HVJ-E initial intratumoral and continuous subcutaneous administration with PD-1 antibody (PD-1 mAb + HVJ-E ip + sc). No clear survival advantage was observed in the PD-1 mAb + HVJ-E ip + sc group (data not shown = patent data). For PD-L1 antibody, the group in which only continuous subcutaneous administration of HVJ-E was added to intratumoral PD-L1 administration (PD-L1 mAb ip + HVJ-E sc) showed significantly longer survival than the group in which only PD-L1 was administered intratumorally without HVJ-E (PD-L1 mAb ip alone) or control. The survival rate was significantly prolonged in the PD-L1 only intratumoral group (PD-L1 mAb) (Figure S2). In other words, intratumoral administration of PD-L1 antibody enhanced the antitumor effect even when only subcutaneous administration of HVJ-E was added. On the other hand, the addition of subcutaneous HVJ-E (PD-1 mAb ip + HVJ-E sc) when PD-1 antibody was administered intrapleurally did not increase survival with subcutaneous HVJ-E administration. In other words, intrapleural administration of PD-1 antibodies did not enhance the antitumor effect by adding only subcutaneous HVJ-E. This may be because PD-L1 is expressed on tumor and dendritic cells, while PD-1 is mainly expressed on cytotoxic T cells. PD-L1 mAb antibodies bind directly to PD-L1 expressed on tumors and inhibit them from evading attack from CTLs. On the other hand, PD-1 mAb antibody targets PD-1 expressed on CTLs and evades the attack of CTLs by tumor antigen. Because of the disseminated lesions in this model, it was thought that intratumoral administration would be more efficient in targeting tumor-expressed PD-L1 than systemic administration of PD-L1 via intraperitoneal administration [30]. In addition, since PD-1 antibodies target PD-1 on CTLs, it may be unlikely that intratumoral administration of PD-1 antibodies would directly affect CTLs that deviate from blood vessels, although the CTLs on which the PD-1 antibodies act are CTLs that deviate from blood vessels, and, in fact, intratumoral administration of PD-1 antibodies has shown little antitumor effect compared to intratumoral administration of PD-L1 antibodies. From these things, we know that major histocompatibility complex (MHC) class-I expression in tumor cells is elevated when HVJ-E is first administered intratumorally and then subcutaneously. This is consistent with the results reported that MHC class-I expression is upregulated during the so-called viral infection.

The downregulation of MHC class-I by treatment with PD-1 or PD-L1 antibodies is one of the mechanisms of tumor resistance to drugs [31], and the fact that the expression of MHC class-I is almost unchanged by PD-1 antibody alone suggests that the MHC class-I expression was once upregulated due to the resistance mechanism. The fact that the expression of MHC class-I is almost unchanged with PD-1 antibody alone suggests that the once elevated MHC class-I may have decreased due to the resistance mechanism. Interferon gamma, produced in T cells, in particular, plays a central role in the expression of antitumor activity, and is also a major driving force in the induction of PD-L1 and MHC class I expression in tumor cells. T cells that are no longer suppressed by PD-L1 by anti-PD-1 antibodies produce interferon-gamma. Normally, this mechanism promotes antigen presentation in target tumor cells and creates an environment conducive to antitumor effects, but in tumor cells with genetic mutations that induce acquired resistance, the signaling system from interferon-gamma becomes completely dysfunctional and the tumor cells themselves are unable to present antigen. In tumor cells with genetic mutations that induce acquired resistance, the antigen-presenting ability of the tumor cells themselves is impaired, and T cells are unable to recognize the tumor cells. Further analysis revealed that there are rare tumors with de novo mutations in the signaling pathway associated with interferon-gamma, which show early resistance to anti-PD-1 [32].

Although CTL processing of tumors proceeds, IFN-γ secreted by CTLs enhances MHC-class I expression and the frequency of TILs, tumor cells become resistant to treatment with PD-1 antibodies [32,33]. We know that MHC class-I expression in tumor cells is elevated when HVJ-E is first administered intratumorally and then subcutaneously (Figure S7). The degree of staining for PD-L1 expression in tumor tissues was also confirmed in the same way as for MHC class-I staining. The expression of PD-L1 in the tumor tissues of the control, CDDP and HVJ-E intratumoral and subcutaneous administration, PD-1 antibody administration, PD-1 antibody and HVJ-E intratumoral and subcutaneous administration, and HVJ-E intratumoral and subcutaneous administration treatment groups was observed by immunohistochemistry (Figure S8). PD-L1 expression was significantly upregulated in the CDDP plus HVJ-E group compared to the control, PD-1 antibody alone, PD-1 antibody plus HVJ-E intratumoral and subcutaneous, and HVJ-E intratumoral and subcutaneous groups. CDDP has been reported to increase PD-L1 expression in vivo and clinically, consistent with the results [34,35].

The staining of this tissue was confirmed at 28 to 31 days after tumor cell inoculation, and since PD-L1 expression in PD-1 antibody therapy to tumor-bearing mouse in vivo was around 9 days after tumor cell inoculation, the expression of MHC class-I also peaked around day 10, similar to PD-L1 expression, and then MHC class-I expression may have peaked around day 10, as was the case with PD-L1 expression, and then decreased due to down-regulation. In any case, MHC class-I expression did not differ from that of the control group. PD-L1 expression was significantly upregulated in the CDDP plus HVJ-E group compared to the control, PD-1 antibody alone, PD-1 antibody plus HVJ-E intrapleural and subcutaneous, and HVJ-E intrapleural and subcutaneous groups. CDDP has been reported to increase PD-L1 expression in vivo and clinically, consistent with the results [34]. Although CDDP enhances MHC class I and PD-L1 expression [35], this ELISpot Compared to Ctrl, there is no significant increase in IFN- with CDDP alone (Figure 4b). Although CDDP + HVJ-E shows a very significant PD-L1 increase in Figure S8, we believe that the presence of elevated PD-L1 indicates that INF-γ is not elevated because it acts as a resistance mechanism against CTLs. The date of staining confirmation of this tissue was 28 to 31 days after tumor cell inoculation, since the peak of PD-L1 expression in PD-1 antibody therapy to tumor-bearing mouse in vivo has been reported in the literature to come relatively early, around 9 days after tumor cell inoculation [36], and then expression may have decreased due to tumor resistance mechanisms. When PD-L1 expression was compared between the PD-1 antibody treatment group and the treatment group with the addition of intrapleural and continuous subcutaneous administration of HVJ-E, the PD-L1 expression was significantly elevated in the combination group, suggesting that treatment with PD-1 antibodies activates the resistance mechanism of the tumor to PD-1 antibodies, resulting in a weakening of PD-L1 expression, which may lead to PD-1 antibody ineffectiveness. In addition, the so-called Cold Status of Tumor Microenvironment is also thought to cause the ineffectiveness of immunotherapy [34,35]. We found that the combination of HVJ-E and PD-1 antibody in tumor tissues enhanced the expression of MHC class I and PD-L1 in a MPM-bearing mouse, and their combined use enhanced the antitumor effect. The combination of chemotherapy and ICIs is currently used in clinical practice for lung cancer and other types of cancer [37], and the safety of HVJ-E has been tested in melanoma and prostate cancer. We will continue to apply this combination therapy (ICIs and chemotherapy) in clinical practice, and in the near future, we believe that the addition of HVJ-E to this combination therapy will improve the anti-tumor effect in actual clinical practice.

## 5. Conclusions

The combination of HVJ-E and chemotherapy or anti-PD-1 antibody significantly prolonged the survival rate of MPM-bearing mouse. In addition to activating CTLs and innate immunity, HVJ-E might have exhibited antitumor effects by maintaining immunogenicity against tumors; activating innate immunity and cytotoxic T cells, and enhancing immunogenicity of tumor tissue. We believe that HVJ-E has the potential to be a beneficial therapy to improve MPM treatment in the future.

## 6. Patents

Patent Cooperation Treaty No. JP2017/039568 (WO2018/084185) is related to the content of this manuscript.

## Data Availability

The data presented in this study would be considered for acquisition upon request to the authors with a rationale.

## Supplementary Materials

The following are available online at https://www.mdpi.com/article/10.3390/cancers15030929/s1, Figure S1: The pleural cavity of mouse 10 days after implanted MSTO-H211 tumor cells, Figure S2: Survival of murine malignant pleural mesothelioma-bearing mouse treated with subcutaneous injection of HVJ-E and intrapleural injection of PD-L1 mAb, Figure S3: Survival of murine malignant pleural mesothelioma-bearing mouse treated with subcutaneous injection of HVJ-E and intraperitoneal injection of PD-1 mAb, Figure S4: Ganglioside analysis of various cell lines. Figure S5: The differences of antitumor effect of HVJ-E according to the frequency of the subcutaneous administration of HVJ-E into human MPM orthotopically implanted mouse, Figure S6: Add-on effect of the subcutaneous administration of HVJ-E into human MPM orthotopically implanted CB-17/SCID mouse, Figure S7: The expression of MHC class-I on tumor tissue of murine MPM tumor-bearing mouse, Figure S8: The expression of PD-L1 on tumor tissue of murine MPM tumor-bearing mouse.
