# Supplementary material for: Add-On Effect of Hemagglutinating Virus of Japan Envelope Combined with Chemotherapy or Immune Checkpoint Inhibitor against Malignant Pleural Mesothelioma: An In Vivo Study
Source: Cancers (Basel). 2023 Feb 1;15(3):929. doi: 10.3390/cancers15030929 (PMC9913709; doi:10.3390/cancers15030929)
Supplement: Supplementary file 1 [file cancers-15-00929-s001.zip › cancers-2108524-supplementary.pdf]

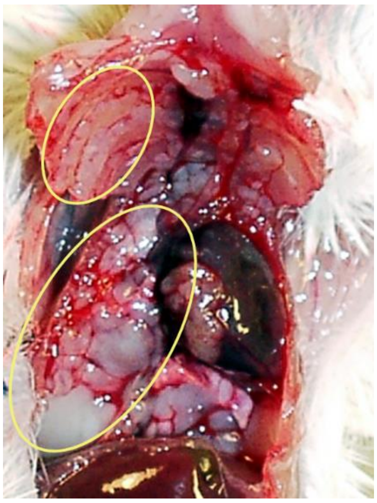

Figure S1. The pleural cavity of mouse 10 days after implanted MSTO-H211 tumor cells. Two million of MSTO-H211 in 50 μL of PBS with 100 μL of Matrigel were administered into the murine parietal pleura confirming the ribs by incised skin of right chest. In the therapeutic experiments, CDDP or HVJ-E was administrated into the affected pleural cavity 8 days after the implantation of MSTO-H211 tumor cells. Ten days after the tumor cells implantation, affected pleural cavity of the control mouse was already filled with the tumor.

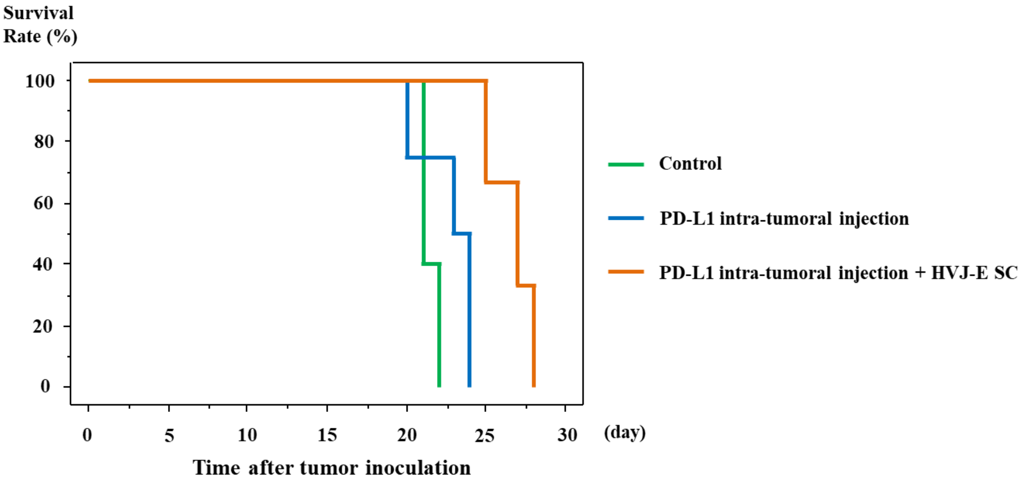

Figure S2. Survival of murine malignant pleural mesothelioma-bearing mouse treated with subcutaneous injection of HVJ-E and intrapleural injection of PD-L1 mAb. Add-on effect of HVJ-E for the intrapleural administration of anti-PD-L1 antibody. Three hundred thousand of AB22G2 cells in 50 μL of PBS with 100 μL of Matrigel were administered into the murine parietal pleura confirming the ribs by incised skin of right chest. Three days after the cell injection, 1500 HAU of HVJ-E was injected into the right pleural cavity and subsequently three subcutaneous injections of HVJ-E were administered every 3 days. These four injections of HVJ-E were considered as one cycle therapy, and this therapy was repeated 2 times maximally. PD-L1 monoclonal antibody was administrated into the peritoneal cavity on the first day of each cycle of HVJ-E treatment (day 3, 17). Three days after the cell injection, 1500 HAU of HVJ-E was injected subcutaneously into the back of the mouse, and subsequently three subcutaneous injections of HVJ-E were administered every 3 days. These four injections of HVJ-E were considered as one cycle therapy, and this therapy was repeated 2 times maximally. PD-L1 mAb was administrated into the peritoneal cavity on the first day of each cycle of HVJ-E treatment (day 3, 17). The mean survival time of the mice that were treated with HVJ-E subcutaneously and PD-L1 mAb intrapleural administration was significantly prolonged compared with that of PD-L1 mAb alone or the control group ($p < 0.05$ respectively).

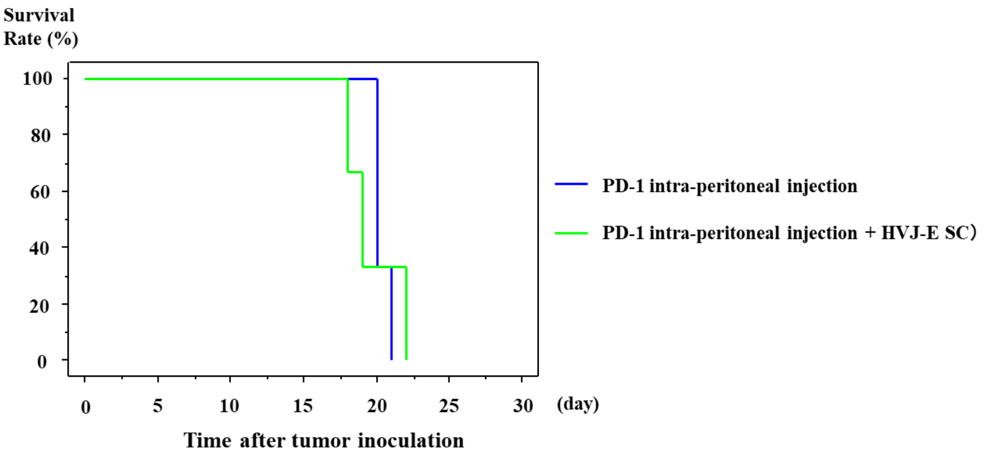

Figure S3. Survival of murine malignant pleural mesothelioma-bearing mouse treated with subcutaneous injection of HVJ-E and intraperitoneal injection of PD-1 mAb. No add-on effect of subcutaneous injection of HVJ-E for the intraperitoneal administration of anti-PD-1 antibody. Three hundred thousand of AB22G2 cells in 50 µL of PBS with 100 µL of Matrigel were administered into the murine parietal pleura confirming the ribs by incised skin of right chest. Three days after the cell injection, 100 µg/100 µl of PD-1 monoclonal antibody was administrated into the peritoneal cavity, or 1000 HAU of HVJ-E was injected subcutaneously into the back of the mouse, and subsequently three subcutaneous injections of HVJ-E were administered every 3 days. Three days after the cell injection, 1000 HAU of HVJ-E was injected subcutaneously into the back of the mouse, and subsequently subcutaneous injections of HVJ-E was administered every 3 days. PD-1 mAb was administrated into the peritoneal cavity on the first day of each cycle of HVJ-E treatment (day 3). There was no significant difference between the mean survival time of the mice that were treated with HVJ-E subcutaneously and PD-1 mAb intraperitoneal administration and that of PD-1 mAb alone.

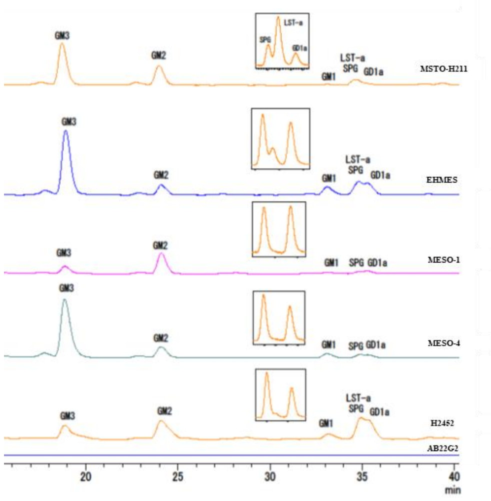

Cell line	Path. Type	GD1a	Sialyl ParaGloboside(SPG)	Ratio
MSTO-H211	Biphasic	2018	4340	3.3
EHMES	Biphasic	23,440	25,491	15.5
MESO-1	Sarcomatoid	6080	5225	12.8
MESO-4	Epithelial	5357	6564	4.7
H2452	Epithelial	40,084	55,223	40.1
AB22G2	Murine	(-)	(-)	

Figure S4. Ganglioside analysis of various cell lines. We determined the quanty of ganglioside for HVJ-E receptors in mesothelioma cells and normal mesothelium using HPLC. From comparison of the positions on the map to the positions of standard acidic PA-oligosaccharides, Peak 1 of 4 cells is predicted to be GM1. Peak 2 from 4 different

cells consisted of SPG, GD1α and LST-a. The expression of ganglioside (GD1α, SPG), a HVJ-E receptor, in various human mesothelioma cell lines was abundant, while AB22, a mouse mesothelioma cell line, showed no expression of the above ganglioside.

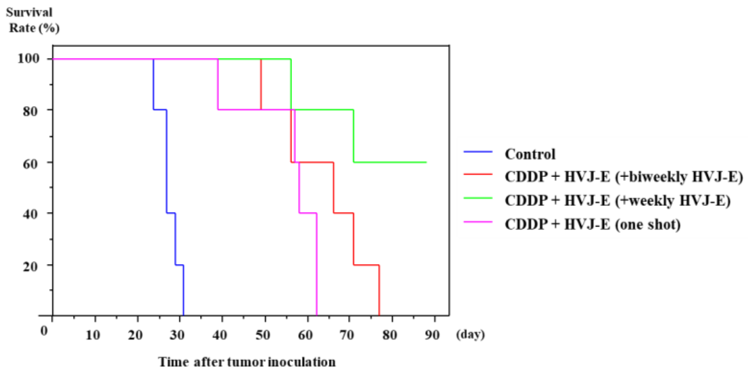

Figure S5. The differences in antitumor effect of HVJ-E according to the frequency of the subcutaneous administration of HVJ-E into human MPM orthotopically implanted mouse. In the MSTO-H211 tumor-bearing SCID mouse model, the difference in survival rates between the groups that received only one intrapleural administration of HVJ-E, those that received one intrapleural administration and continued weekly subcutaneous administration, and those that received one intrapleural administration and continued subcutaneous administration every two weeks was investigated, and all HVJ-E treatment groups showed that the survival rate was significantly prolonged in all HVJ-E-treated groups compared to the control group ($n = 5$) ($p < 0.005$).
The group that received intrapleural administration followed by weekly subcutaneous administration showed significantly prolonged survival compared to the group that received only intrapleural administration ($p < 0.05$). On the other hand, there was no significant difference between the group that received intrapleural administration followed by subcutaneous administration once every two weeks and the group that received only intrapleural administration ($p = 0.183$). Although there was no significant difference between the group that received intrapleural plus weekly subcutaneous administration and the group that received intrapleural plus bi-weekly subcutaneous administration, there was a tendency toward a longer survival rate with weekly subcutaneous administration ($p = 0.06$).

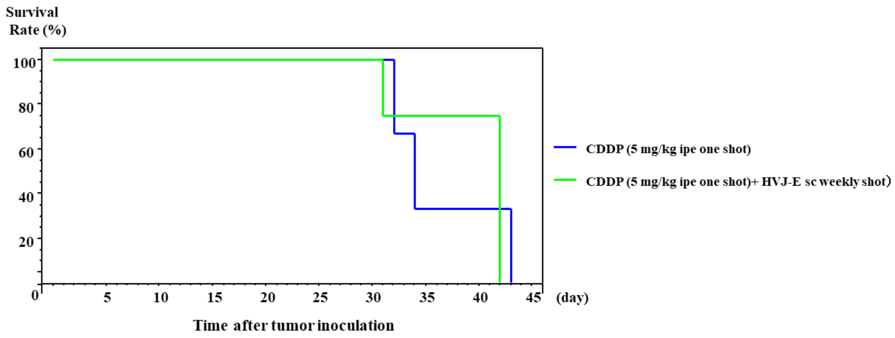

Figure S6. Add-on effect of the subcutaneous administration of HVJ-E into human MPM orthotopically implanted CB-17/SCID mouse. Two hundreds thousand cells of MSTO211H cell line/50 μL PBS with 100 μL of Matrigel will be injected into the murine pleural cavity. Nine days after tumor cell injection, 5 mg /kg of CDDP was injected into the peritoneal cavity, and 1000 HAU of HVJ-E was injected subcutaneously weekly ($n = 4$). In the SCID mouse

tumor-bearing model, the synergistic effect on cisplatin was not observed when only weekly subcutaneous administration of HVJ-E was added without intratumoral administration of HVJ-E, suggesting that intra-tumoral administration of HVJ-E is necessary in this mouse model MPM (p = 0.935)

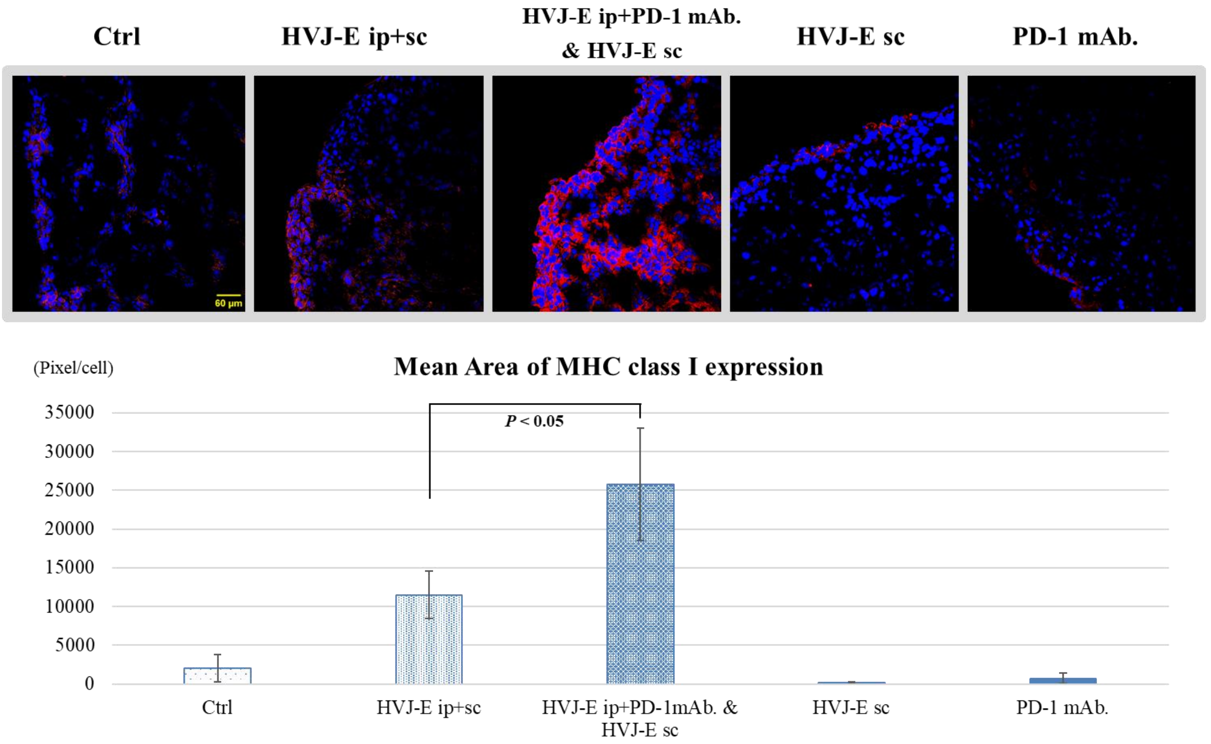

Figure S7. The expression of MHC class-I on tumor tissue of murine MPM tumor-bearing mouse. The degree of staining for MHC Class-I expression in tumor tissues was confirmed by immunohistochemistry. The expression of MHC Class-I in the tumor tissues of the control, cisplatin and HVJ-E intratumoral and subcutaneous administration, PD-1 antibody administration, PD-1 antibody and HVJ-E intratumoral and subcutaneous administration, and HVJ-E intratumoral and subcutaneous administration treatment groups was observed by immunohistochemistry. The number of tumor cells was calculated from the number of Hochest staining dots, and the area of MHC Class-I staining per tumor cell was quantified.

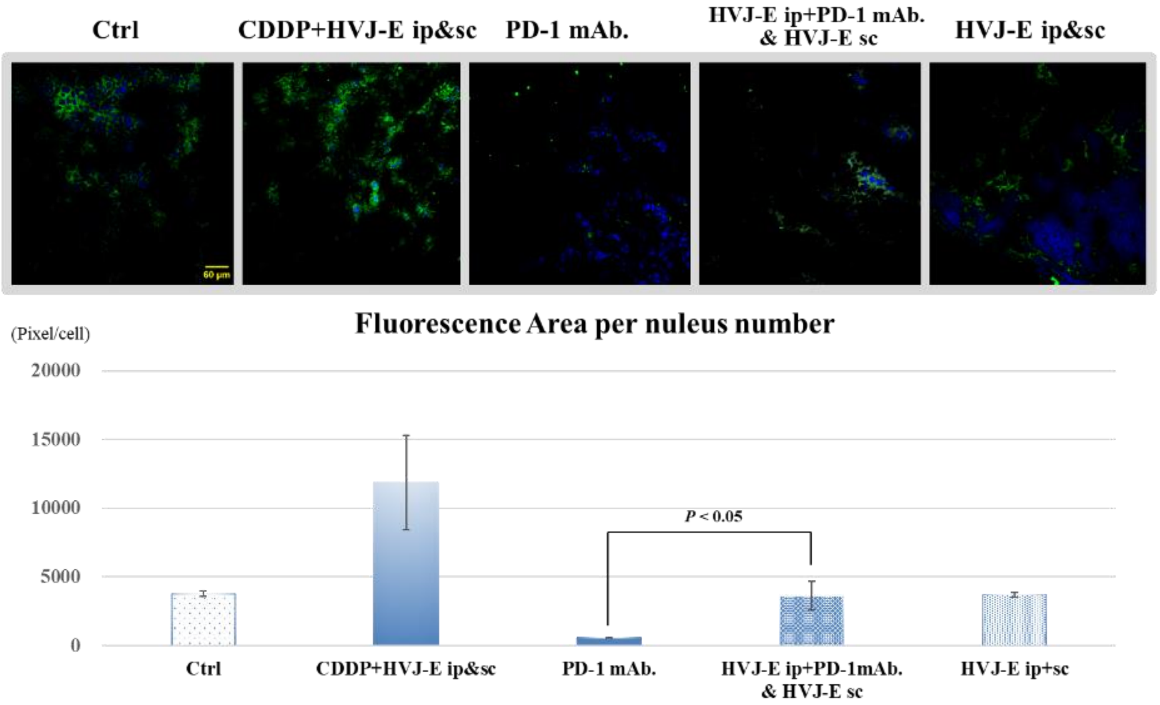

Figure S8. The expression of PD-L1 on tumor tissue of murine MPM tumor-bearing mouse. The degree of staining for PD-L1 expression in tumor tissues was also confirmed in the same way as for MHC class-I staining. The expression of PD-L1 in the tumor tissues of the control, cisplatin and HVJ-E intratumoral and subcutaneous administration, PD-1 antibody administration, PD-1 antibody and HVJ-E intratumoral and subcutaneous administration, and HVJ-E intratumoral and subcutaneous administration treatment groups was observed by immunohistochemistry. The number of tumor cells was calculated from the number of Hochest staining dots, and the area of PD-L1 staining per tumor cell was quantified.
